# Cryopreservation preserves the morpho-structural and mechanical integrity of human decellularized tracheas and supports a reduced immunogenic profile

**DOI:** 10.3389/fbioe.2026.1724094

**Published:** 2026-05-14

**Authors:** Elena Stocco, Silvia Barbon, Alessia Cardaci, Antonia Barbazza, Marta Confalonieri, Martina Contran, Valentina Manzo, Silvia Todros, Piero G. Pavan, Raffaele De Caro, Veronica Macchi, Andrea Porzionato, Diletta Trojan

**Affiliations:** 1 Department of Neuroscience, Section of Human Anatomy, University of Padova, Padua, Italy; 2 Department of Women’s and Children’s Health, University of Padova, Padua, Italy; 3 Department of Surgery, Oncology and Gastroenterology, University of Padova, Padua, Italy; 4 Foundation for Biology and Regenerative Medicine, Tissue Engineering and Signaling - T.E.S. Onlus, Padua, Italy; 5 Fondazione Banca dei Tessuti del Veneto, Treviso, Italy; 6 Department of Industrial Engineering University of Padova, Padua, Italy; 7 Tissue Engineering Lab, Fondazione Istituto di Ricerca Pediatrica Città della Speranza, Padua, Italy

**Keywords:** cryopreservation, decellularization, mechanical behaviour, reduced immunogenicity, trachea

## Abstract

**Background:**

A suitable tracheal substitute must support revascularization and regeneration, while resisting necrosis and infection. Decellularized tracheas are promising candidates; however, degradation limits their shelf life, making tissue banking essential. Although various decellularization methods have been developed, few studies assess the impact of storage, particularly cryopreservation, on tissue quality, limiting their clinical translation.

**Methods:**

In this study, decellularized tracheas and decellularized + cryopreserved tracheas were developed from three human donors, segmented and processed accordingly. These were compared to native tracheas in terms of macroscopic appearance and structural integrity. Thus, residual nuclei/DNA were assessed using DAPI staining and DNA quantification. Histological stains (Hematoxylin and Eosin, Alcian Blue, Masson’s Trichrome, Weigert Van Gieson) assessed tissue and extracellular matrix architecture. Glycosaminoglycans and elastic fibers were quantified through both quantitative and semiquantitative methods. Immunostaining and semi-quantification for Human Leukocyte Antigen–DR (HLA-DR) was performed to preliminarily evaluate residual immunogenicity reduction. Ultrastructure and mechanical properties were analyzed using scanning electron microscopy and compression tests.

**Results:**

The decellularization protocol effectively reduced DNA content to <50 ng/mg, confirmed after cryopreservation. Native tracheas showed normal respiratory epithelium, whereas decellularized tracheas and decellularized + cryopreserved tracheas retained only the basal lamina. Submucosa was similar across groups, except for the absence of nuclei in treated samples. Glycosaminoglycans and collagen were well preserved, as showed by Alcian Blue and Masson’s trichrome stainings. Elastic fibers integrity and content were reduced in accordance with Weigert Van Gieson staining and morphometric analysis. Only few HLA-DR immuno-positive elements were recognized after treatments, as confirmed by semi-quantitative analysis. Scanning electron microscopy revealed epithelial cell removal with preserved basal lamina and adventitial collagen, although less compact. Treated cartilage showed empty lacunae. Mechanical testing revealed no significant differences in stiffness between groups.

**Conclusion:**

Study results indicate that combining decellularization with cryopreservation effectively preserves tracheal structure and further reduces HLA-DR–immunopositive elements compared with decellularized trachea. These findings support the clinical potential of decellularized + cryopreserved grafts for tracheal replacement.

## 1 Introduction

To date, recovering from extensive tracheal damage remains a significant challenge in respiratory medicine. While short-length defects ascribable to trauma or benign/malignant diseases can often be addressed through end-to-end anastomosis, lesions that involve more than half the tracheal length in adults or more than a third in children typically would require tracheal replacement (Grillo, 2002; Wei et al., 2024; Delaere et al., 2025). However, despite several repair strategies being attempted, none have yielded satisfactory outcomes (Etienne et al., 2018): allografts implantation requires lifelong immunosuppression; mechanical devices/prostheses do not restore tissue function. Moreover, artificial implants have a limited lifespan and may trigger allergic reactions as consequence of material abrasion (Law et al., 2016). Other issues including tissue ongrowth, stenosis, narrowing of the lumen with bleeding have been also frequently reported (Tseng et al., 2024). Consequently, severe tracheal lesions are typically managed with stent placement, which can maintain lumen patency but does not provide a definitive treatment for the patient (Tseng et al., 2024). Complications such as stent migration, mucus plugging, granulation tissue formation, cough, stent rupture, and infection are reported (Chen et al., 2024). Failure of conventional therapies has prompted research into tissue engineering; this strategy has been recognized as one of the most promising approaches for clinical application in case of long-segment tracheal damages (He et al., 2012; Etienne et al., 2018).

In recent years, a clinically oriented strategy has attracted increasing attention that involves the use of cryopreserved aortic allografts as airway substitutes, combined with stent support and muscle flap coverage. The aorta has a diameter comparable to that of the trachea and a robust structural integrity; it is also elastic and resistant to infection (Wei et al., 2024). These features make it a particularly attractive option. Moreover, this approach, which avoids the need for decellularization, has demonstrated encouraging preclinical and clinical outcomes, representing a promising strategy for tracheobronchial replacement (Martinod et al., 2022; Martinod et al., 2025). However, aortic matrices are not airway-specific tissues; it descends that their functional integration is strictly related to progressive remodeling, epithelialization, and partial cartilaginous metaplasia, processes that are host-driven and thus variable and difficult to understand comprehensively. Moreover, long-term airway stability generally requires a period of stent placement to avoid the risk of collapse (Martinod et al., 2013; Martinod et al., 2022; Wei et al., 2024). These considerations have prompted interest in orthotopic allografts.

An ideal tracheal scaffold is expected to show a tubular shape resembling native trachea, biocompatibility, biodegradability, a certain porosity thus allowing cell ingrowth and ECM deposition, adequate mechanical behaviour and flexibility. *In vivo*, it must undergo revascularization while supporting tissue regeneration and discouraging graft necrosis or infection (Xu et al., 2017; Zhang et al., 2022). In consideration of this, the use of a decellularized trachea as a scaffold for tissue regeneration may represent an appealing strategy (Stocco et al., 2023a). Together with physical support for cell adhesion, it also promotes cells migration, proliferation, and differentiation (Ghosh and Pati, 2023).

Revising the literature, trachea decellularization has been achieved using a combination of chemical and physical strategies, as well as chemical and enzymatic treatments coupled with physical methods (Stocco et al., 2023a). Epithelium and glands of the mucosa/submucosa are the major antigenic structures of the trachea (presence of Major Histocompatibility Complex class I (MHC-I) and class II (MHC-II) antigens); conversely, the cartilage compartment is recognized as an immune-privileged area (absence of MHC-I and MHC-II) also due to dense matrix and avascularity (Bujia et al., 1990; Nakanishi, 2009; Shaari et al., 1998; Zeng et al., 2023). Accordingly, the antigenicity of tracheal grafts can be significantly reduced by removing the epithelium and mixed glands, which are the main responsible of rejection following tracheal allotransplantation (Liu et al., 2000). The mucosal layer, rather than the cartilage, constitutes the predominant antigenic component responsible for graft rejection (Liu et al., 2000). However, like as for other human tissues (Porzionato et al., 2018), there is no gold-standard method for trachea decellularization: the adopted protocol is required to efficiently remove cells while preserving extracellular matrix (ECM) architecture. This is crucial to maintain organ patency and favourable mechanical behaviour which are encountered among the most critical challenges in trachea tissue engineering (Boazak and Auguste, 2018). Furtherly, compliance with the “Guide to the quality and safety of tissues and cells for human application” (EDQM, European Directorate for the Quality of Medicines and Healthcare - www.edqm.eu/en/guide-to-the-quality-and-safety-of-tissues-and-cells-for-human-application) is fundamental; the protocol should be reproducible in classified environments and feasible using reagents suitable for human use.

Biological scaffolds are susceptible to degradation whether stored in buffer solutions (Zouhair et al., 2019). Therefore, tissue banking is fundamental to permit off-the-shelf availability of the decellularized tissues and to facilitate their routine use in clinical practice (Zouhair et al., 2019). Certainly, the preservation method must be ECM respectful, without showing detrimental effects over its composition and ultrastructure. To date, despite many decellularization strategies have been reported and compared (Stocco et al., 2023a), only few studies focused on the impact of storage conditions over the scaffolds’ characteristics. This, in turn, limits their clinical use (Urbani et al., 2017) and represents a significant gap in the path leading to their choice for implant, as afflicting constructs quality, efficacy and safety (Freitas-Ribeiro et al., 2022). Furthermore, protocols developed so far often do not fully comply with the regulatory requirements applicable to tissue banks, relying primarily on literature revision. This represents an additional limitation to their clinical applicability (Porzionato et al., 2018).

Among long-term preservation strategies, cryopreservation is broadly used, being proposed for cryopreserved aortic allografts (currently representing the only tracheobronchial replacement strategy), supported by preclinical and prospective clinical data (Seguin et al., 2009; Makris et al., 2010; Martinod et al., 2022; Martinod et al., 2025) and proposed also for tracheal grafts (Yokomise et al., 1996). Cryopreservation consists in soaking biological tissues in a cryoprotectant solution and maintaining them at a temperature between −140 °C and −196 °C (in liquid nitrogen). Under these conditions, all chemical reactions, biological processes and physical intra- and extracellular activities are interrupted; ideally, a cryopreserved organ can be kept indefinitely (Bakhach, 2009). Interestingly, previous studies have shown that cryopreservation not only preserves organs and tissues, maintaining ECM organization and mechanical properties, but also reduces immunogenicity, thereby lowering the risk of immune rejection (Wang et al., 2021). Yokomise et al. (1996), storing fresh trachea in a deep freezer at −85 °C for 8–10 months, confirmed these findings. Thus, ideally, combining decellularization with cryopreservation may not only guarantee optimal tissue preservation (cryopreservation is a well-established method for storing human tissues intended for transplantation, as recognized by European guidelines and required by Italian regulations), but also further improves the outcomes of decellularization (Stocco et al., 2023b).

In this study, involving a synergic collaboration between the Tissue Bank *Fondazione Banca dei Tessuti del Veneto–FBTV* (Treviso, Italy) and the Section of Human Anatomy of the University of Padova (Padua, Italy), we developed and compared two human-derived trachea substitutes: decellularized trachea versus decellularized + cryopreserved trachea. In addition to evaluating the effectiveness of the decellularization protocol, we also assessed the impact of cryopreservation on the characteristics of decellularized trachea.

## 2 Materials and methods

### 2.1 Trachea isolation, microbiological analyses and sampling

Three human tracheal samples were collected by FBTV from human deceased donors, after obtaining proper consent from the next of kin, according with the Italian national regulation on tissue banking. Tracheal samples unsuitable for transplantation but morphologically unaltered were utilized. Retrievals were performed within 24 h of cardiac arrest or 12 h if the cadavers were not refrigerated during the first 6 hours after death. The tracheas were harvested from the larynx to the lungs, including a minimum of twelve tracheal rings. Thus, specimens were placed in antibiotic solution made up with BASE medium (Alchimia srl, Italy), gentamicin 200 μg/mL (Fisiopharma, Italy), vancomycin 100 μg/mL (Pharmatex, Italy) and meropenem 200 μg/mL (Fresenius Kabi AG, Germany) (this solution was validated for tissue decontamination (Serafini et al., 2016; Paolin et al., 2018; Montagner et al., 2024a)), and transported to FBTV at +2 °C/+8 °C. Basic serological and molecular analyses were then conducted according to FBTV standard operating procedures. Upon receiving negative serology results, samples’ processing started.

Microbiological tests were established throughout the tracheal tissue processing to verify the compliance with the acceptance criteria and regulation. Tissue samples were tested during processing and at the end of decellularization before cryopreservation. Liquid samples of the solutions that were in contact with the tissue during processing were inoculated and incubated in BD BACTEC culture vials, in accordance with the manufacturer’s instructions (BD, Becton, Dickinson and Company, United States). Tissue samples after retrieval and after processing were inoculated and incubated in Trypcase Soy Broth and Clear-Thio Broth, in accordance with the manufacturer’s instructions (Biomerieux SA, France). If the samples tested positive, the microorganisms were isolated and identified using standard procedures. Finally, environmental microbiological monitoring was conducted according to EDQM guide at each step of the process using the following plates: Tryptone Soya Agar irradiated and TSA contact neutral TLHT (VWR, Belgium).

Preliminarily, trachea samples (n = 3 donors) were carefully rinsed through multiple washes in saline solution and then divided into three segments, each containing four rings. Each segment was assigned to a different experimental condition: native trachea (NativeT, control group), decellularized trachea (DecellT), and decellularized and cryopreserved trachea (DecellT + CryoT). The rings within each segment were subsequently allocated to different characterization analyses, including 4,6-Diamidino-2-Phenylindole (DAPI) nuclear staining, DNA content analysis, histological and immunohistochemical analyses, glycosaminoglycan (GAG) quantification, and scanning electron microscopy (SEM). The samples were placed in separate sterile plastic containers, soaked in the decontamination solution previously described and stored at +2 °C to +8 °C for 48 h prior to proceeding with the following analyses/protocols, as described below.

### 2.2 Trachea segments processing

#### 2.2.1 Decellularization

Tracheal decellularization was performed over three consecutive days following a patented protocol (Italian patent number; IT202200003260A1 – Ufficio Italiano Brevetti e Marchi; Title: “Metodo per la preparazione di derma decellularizzato in condizioni asettiche” available on *Google Patents.* Inventor: Diletta Trojan, Giulia Montagner). The protocol relies on a hypertonic solution (sodium chloride, NaCl) and two reagents—benzonase (Sigma-Aldrich, United States) and sodium cholate (Sigma-Aldrich). Specifically, a first incubation was performed in 6% (w/v) NaCl at 37 °C overnight, followed by a second incubation in an aqueous benzonase solution (10 U/mL) for 20 h at 37 °C; this was followed by incubation in an aqueous sodium cholate solution (0.5%) for 20 h at room temperature (RT). After completion of the decellularization process, the tracheal rings were incubated overnight in the previously described antibiotic solution. All the procedures were conducted under aseptic conditions and in accordance with current regulations on human allograft banking. Sample handling was carried out in cleanrooms using a Class II biosafety cabinet.

#### 2.2.2 Cryopreservation

To develop DecellT + CryoT samples, cryopreservation process followed that of decellularization.

Following decellularization, the tissues (named DecellT + CryoT) were placed in sterile cryo-resistant bags made of ethylene vinyl acetate (Agricons Ricerche, Italy) in a preservation solution of BASE medium (Alchimia Srl), 10% dimethyl sulfoxide (DMSO) (WAK-Chemie Medical GmbH, Germany) and 10% human albumin (Kedrion S. p.A., Italy). Subsequently, the samples were transferred into a programmable cryogenic freezer (Planer KryoSave Integra 750–30, Planer Limited, United Kingdom), which triggered a controlled cooling rate. Specifically, the freezing program lasted 90 min and involved a gradual decrease in temperature until reaching −140 °C. Upon completion of the freezing phase, the samples were stored for 1 month in a dedicated tank under nitrogen vapor at −160 °C at FBTV. The temperature range complies with the “Guide to the quality and safety of tissues and cells for human application” (EDQM, European Directorate for the Quality of Medicines and Healthcare).

### 2.3 Trachea segments characterization

#### 2.3.1 Nuclei detection by fluorescence staining

To verify the presence and distribution of cells’ nuclei within tracheal cartilage, the DecellT and the DecellT + CryoT specimens were compared with the NativeT ones using fluorescence analysis with DAPI; preliminarily, DecellT + CryoT were slowly thawed submerging the packaging in a water bath at 37 °C for 15–20 min. Once thawed, the trachea grafts were washed in PBS for 3–5 min to elute the cryoprotectant.

Three rectangular specimens/patient, one for each condition, were embedded in Tissue Freezing Medium (Leica Biosystems, Nussloch, Germany) and frozen prior to being cut into 10–12 µm thick sections using a cryomicrotome (Leica CM 1850 UV; Leica Microsystems, Wetzlar, Germany). Hence, the sections were acetone-fixed, mounted with Vectashield mounting medium for fluorescence with DAPI (Vector Laboratories, Burlingame, CA, United States) and photomicrographs were acquired with a Leica LMD6 (Leica Microsystems) connected to a Leica DFC320 high-resolution digital camera (Leica Microsystems) and a computer equipped with software for image acquisition (LasX, Leica Microsystems). Once mounted, the sections were placed in a dark environment for a brief incubation period before being examined.

#### 2.3.2 Quantitative assessment of genetic material residues: DNA quantification

To assess immunogenic material content in DecellT and DecellT + CryoT, residual DNA was extracted by the DNeasy Blood and Tissue Kit (Qiagen, Düsseldorf, Germany). Three rectangular specimens/patient for each condition, weighting approximately 13 mg, were lysed with Proteinase K (Merck Life Science) at 56 °C ON and the lysates were then loaded onto the DNeasy Mini spin columns allowing for selective total DNA purification. Qubit 4 fluorometer and kit (ThermoFisher Scientific, Waltham, MA, United States) were used for eluted DNA fluorometric quantification; absorbance was measured at 260 nm and results were normalized to tissue weight (i.e., ng DNA/mg tissue). NativeT samples were used as control.

DNA was measured in full-thickness tracheal tissue to provide a comprehensive evaluation of all tissue components, including both cartilage and soft tissues. Results were expressed as ng/mg of wet tissue.

#### 2.3.3 Histological analyses

NativeT, DecellT, and DecellT + CryoT samples were fixed in 10% formalin, paraffin-embedded, and sectioned into 5 μm thick slices for histological analysis, following standard laboratory protocols (Stocco et al., 2023b). Haematoxylin and eosin staining was performed to visualize cell nuclei (hematoxylin, deep blue-purple) and overall tissue organization (eosin, pink). Alcian blue staining was used to detect acidic mucins and glycosaminoglycans (GAGs) (blue). Masson’s Trichrome staining (Masson trichrome staining kit; Bio-Optica, Milano, Italy) was applied to evaluate connective tissue, with a particular focus on collagen distribution (green). Weigert Van Gieson staining was performed to visualize elastic fibers (black), using a Weigert Staining Kit for Elastic Fibers-Rapid Method (Bio-Optica) according to the manufacturer’s instructions.

A minimum of three rectangular specimens/patient were considered for each condition.

#### 2.3.4 Morphometric study for elastic fibers quantification

Elastic fibers content within the mucosa/submucosa and adventitia sides were quantified using ImageJ software (version 1.53c, U.S. National Institutes of Health, Bethesda, Rockville, MD, United States), following a previously described method (Stocco et al., 2023b). Briefly, purplish-coloured regions were identified from images captured in bright field at ×40 magnification and saved as TIFF files. The areas of interest were determined by examining histograms of hue, saturation, and brightness distributions, and appropriate thresholds were applied for each: hue, 127–225; saturation, 0–255; and brightness, 0–185. All images were analyzed using the same thresholds. Results are presented as percentage areas stained in purple out of the total area of the acquisition field.

A total of six different fields per section (3 from mucosa/submucosa and 3 from adventitia) from each donor/group were evaluated.

#### 2.3.5 Biochemical assay for GAGs quantification

Presence of GAGs within the NativeT, DecellT and DecellT + CryoT was determined using the Chondrex Inc. Glycosaminoglycans Assay Kit (DBA Italia S. r.l., Milan, Italy). About 10 mg of tissue samples (three samples/group for each donor) were digested in papain solution at 60 °C ON for GAG solubilisation. Hence, solubilized GAGs were labelled with the cationic dye 1,9 dimethylmethylene blue (DMB) and colorimetric reaction was read at 530 nm by using the Microplate auto reader VICTOR3™ (PerkinElmer, Waltham, MA, United States).

GAGs were quantified in full-thickness tracheal samples to provide an integrated assessment of both cartilage and soft tissue compartments. Results were expressed as ng/mg of wet tissue.

Three samples/patient were considered for each condition.

#### 2.3.6 Human Leukocyte Antigen immunolocalization and immunoreactivity semi-quantification

The presence of MHC class II (HLA-DR) antigens is associated with sample immunogenicity and was evaluated as a preliminary assessment of reduced tracheal immunogenicity following decellularization and decellularization + cryopreservation. Anti-HLA-DR (monoclonal mouse anti-HLADR antigens, M0746, Dako) was diluted 1:100 in PBS; epitope retrieval was performed with 10 mM sodium citrate buffer, pH 6.0, at 77 °C for 15 min. Sections were then incubated with peroxidase-blocking serum (EnVision FLEX Peroxidase-Blocking Reagent; Dako) for 5 min in order to avoid unspecific binding before incubation for 1 h at room temperature (RT) with the above primary antibody. Specific binding of the primary antibody was revealed by incubation with the secondary antibody (EnVision FLEX Mouse-Linker and EnVision FLEX Rabbit-Linker; Dako) for 15 min and EnVision FLEX/HRP polymer for 20 min. Subsequently, 3,30-diaminobenzidine (EnVision FLEX Substrate Buffer + DAB + Chromogen; Dako) was used to highlight the positivity of the reaction. Finally, the sections were counterstained with haematoxylin. NativeT samples were used as positive controls for marker expression, whereas negative controls were prepared by incubating sections without primary antibodies. A Dako Autostainer/Autostainer Plus (Dako, Milan, Italy) was used.

At least three specimens/patient were considered for each experimental condition.

Immunoreactivity was semi-quantified by ImageJ software (http://rsb.info.nih.gov/ij/). Three different fields/sections were analyzed to determine immunopositivity in the whole thickness. Images previously acquired in bright field (5× magnification) were saved as TIFF files, converted in 8 bit and adjusted on brightness/contrast parameters. Hence, immunoreactive percentage area was measured after threshold determination, adjusting manually the intensity range in order to avoid the measurement of non-reactive particles (Stocco et al., 2024).

### 2.4 Ultrastructural analysis with scanning electron microscopy

Scanning Electron Microscopy (SEM) allowed for ultrastructural characterization of the samples. NativeT, DecellT, and DecellT + CryoT rectangular specimens (n = 3 for each condition and donor) were compared to assess potential differences in the appearance of the respiratory epithelium, the adventitial side, and the cartilaginous component. Briefly, the samples were placed in a 24-well multiwell plate and soaked in 2.5% glutaraldehyde solution in 0.2 M phosphate buffered saline (PBS) (pH 7.4) for at least 72 h. Thus, the samples were dehydrated with a graded ethanol series (from 30% to 100%), exposed to critical-point drying and gold sputtering and finally observed by a SEM system JSM-6490 (Jeol United States, Peabody, MA, United States).

### 2.5 Compressive mechanical test

**FIGURE 1:**
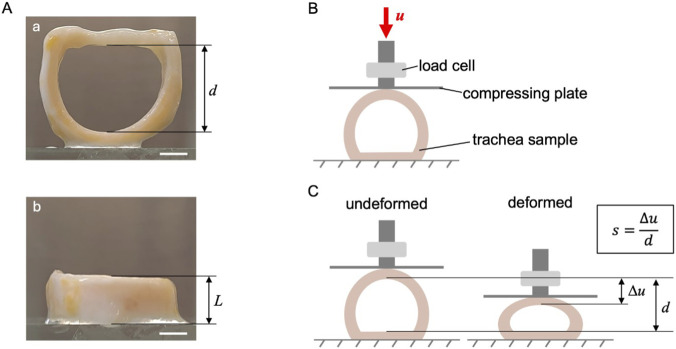
**(A)** Example of a trachea sample for mechanical testing, consisting of three cartilaginous rings, with indicated the proximal-distal diameter *d* **(a)** and length of the segment *L* **(b)**. Scale bars: 5 mm. **(B)** Scheme of the experimental set up for the compression test. A flat plate compresses the sample along the distal-proximal direction, with a displacement *Δu*. **(C)** Definition of plate displacement *Δu*, initial diameter *d*, and compressive deformation along the distal-proximal axis *s,* with reference to undeformed and deformed trachea samples.

The mechanical compressive behaviour of human tracheas was analysed to evaluate the impact of decellularization, eventually combined with cryopreservation, on the organ mechanical properties. For each donor (n = 3), one sample of three rings was tested in its native state (NativeT), one after decellularization (DecellT), and one after both decellularization and cryopreservation (DecellT + CryoT). Hence, for each condition (NativeT, DecellT and DecellT + CryoT), three segments of three rings-each were considered. Prior to testing, geometrical parameters (i.e., proximal-distal diameter d and the length L (Figures 1A,a,b respectively) were measured through sample image analysis with ImageJ2 software (Rueden et al., 2017). Mechanical tests were performed on the Bose ElectroForce® Planar Biaxial Test Bench instrument (TA Instruments, New Castle, DE, United States) under displacement control, employing a 22 N load cell. Each sample was placed on the lower compression plate with the muscular tunic facing downward and fixed with cyanoacrylate adhesive. The upper plate, parallel to the lower one, was displaced downward at a constant rate of 0.2 mm s^-1^ until a 50% reduction in the initial proximal-distal diameter was achieved (Figure 1B). To ensure that the mechanical data were not biased by sample-specific dimensions, the results were processed to remove the influence of each sample’s individual diameter and length. Specifically, compressive deformation along the distal-proximal axis *s* (Figure 1C) was defined as the ratio between the plate displacement *Δu* and the initial diameter of the sample in the distal-proximal direction *d*, which differed among samples. Moreover, to enable dimension-independent comparison, the force per unit length *f* (N/mm) was calculated by dividing the measured force *F* by the specimen length *L*, also varying from sample to sample. The secant modulus *k* was defined as the slope of the linear portion of the force–deformation curve between 20% and 50% of compressive deformation and used to estimate the sample stiffness for an ovalization deformation.

### 2.6 *In vitro* cytotoxicity

#### 2.6.1 HM1SV40 cell cultures

Immortalized human bone marrow cells HM1-SV40 (De Angeli et al., 2004) were used for *in vitro* cytotoxicity assay. Preliminarily, the cells were cultured in proliferation medium consisting in Alfa-Modified Eagle Medium (α-MEM) Without Nucleosides (ThermoFisher Scientific) added in 16.5% fetal bovine serum (FBS) (ThermoFisher Scientific), 1% glutamine and 1% penicillin/streptomycin solution (100 mg/mL). During *in vitro* expansion, cells were maintained at 37 °C, 5% CO_2_ and 95% humidity; after 4 days of culture, cells were detached using a trypsin–EDTA solution, counted by Trypan Blue exclusion using an automated cell counter (TC20 Automated Cell Counter, Bio-Rad, Hercules, CA, United States), and seeded into 24-well plates at a density of 10,000 cells/cm^2^. After seeding, cells were allowed to adhere for 24 h before performing the cytotoxicity test.

#### 2.6.2 Culture medium conditioning

The release of decellularization and/or cryopreservation residues which may have detrimental effects over cells/tissue following implantation, was assessed by the cytotoxicity extract test (Barbon et al., 2020; Barbon et al., 2022a; Barbon et al. 2022b). Samples of DecellT and DecellT + CryoT were incubated in HM1-SV40 cell proliferation medium (100 mg of tissue per milliliter) for 72 h at 37 °C, 5% CO_2_ and 95% humidity. Conditioned media were then used to culture HM1-SV40 cells seeded on 24-well plates as previously described.

#### 2.6.3 Cell viability assay

HM1-SV40 cells were incubated with conditioned media for 24 and 72 h. Untreated cells served as negative control, while cells exposed to cytotoxic conditions (50% dimethyl sulfoxide, DMSO) were used as positive control. Following the incubation periods, cell viability, proliferation and cytotoxicity were assessed through (3-[4,5-dimethylthiazol-2-yl]-2,5 diphenyl tetrazolium bromide) MTT assay, which measures cellular metabolic activity. Briefly, the conditioned culture medium was removed and replaced with basal medium (α-MEM Without Nucleosides) supplemented with 0.5 mg/mL of MTT. Following the incubation period, the MTT solution was removed and the violet MTT-formazan products were extracted with acidified isopropyl alcohol (0.04 M HCl in isopropyl alcohol) (Carlo Erba, Milan, Italy) through gentle stirring at room temperature, for 15 min. Formazan solutions optical density was read at 570 nm by analyzing samples with a VICTOR3™ Microplate auto reader (PerkinElmer, Waltham, MA, United States). Results were expressed as percentage of cell viability in treated samples versus the untreated negative control (100% viability).

### 2.7 Statistical analysis

Data are presented as mean ± standard deviation (SD) from at least three replicates. Statistical analyses were performed using the Kruskal–Wallis nonparametric one-way ANOVA, followed by appropriate *post hoc* comparisons. Differences were considered statistically significant at *p* < 0.05.

Statistical analysis was performed using GraphPad Prism version 8.4.2 for Windows (GraphPad Software, San Diego, CA, United States).

## 3 Results

### 3.1 Microbiological assessments results

All the environmental controls were negative, demonstrating that the entire process is achievable in a Good Manufacturing Practice (GMP) compliant facility and in accordance with tissue bank practice. All the microbiological tests were compliant with the acceptance criteria. Tracheal samples were effectively decontaminated, even without terminal sterilization.

### 3.2 Tracheas macroscopic appearance, fluorescence characterization with DAPI and DNA content

NativeT distinguished for a pink-red colour, typical of the fresh tissue after dissection; the cartilage rings were consistent and the pars-membranacea appeared as smooth and well-stretched.

**FIGURE 2:**
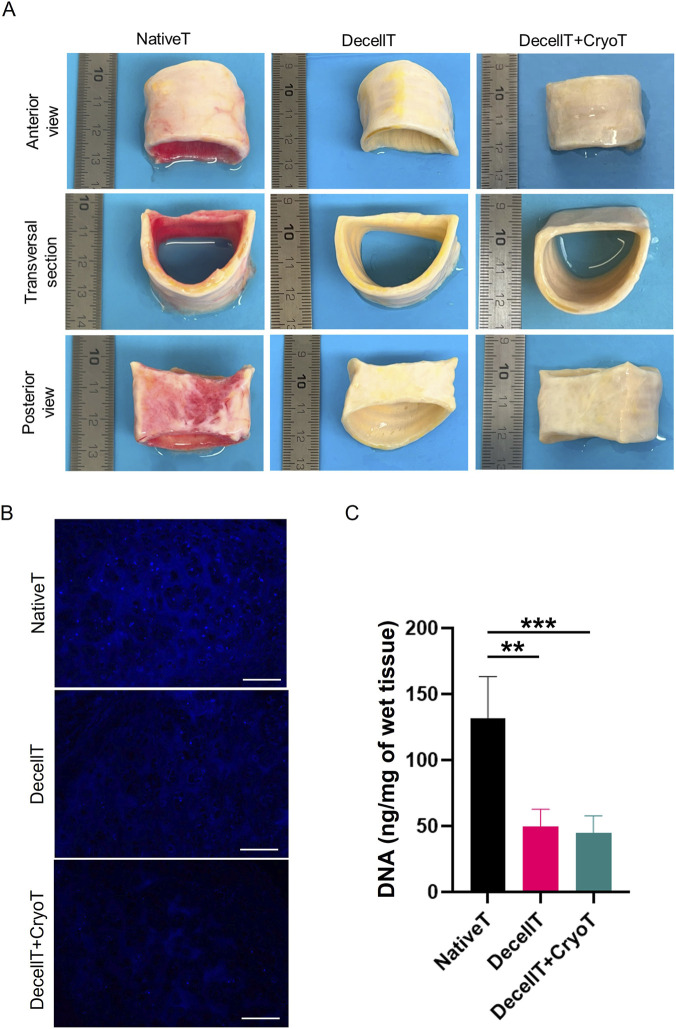
**(A)** Macroscopic appearance of native trachea (NativeT), decellularized trachea (DecellT) and decellularized + cryopreserved trachea (DecellT + CryoT). **(B)** DAPI-staining showing as blue dots the presence of nuclei within cartilage matrix of NativeT, DecellT and DecellT + CryoT (scale bar: 200 µm). **(C)** DNA quantification graph showing DNA (ng/mg of wet tissue) content within the cohort (^**^p < 0.01; ^***^p < 0.001).

Following decellularization the tissue turned to a whitish colour, also maintained after cryopreservation. Decellularization, whether alone or combined with cryopreservation, did not affect lumen patency and no collapse-related signs were observed (Figure 2A).

In parallel, DAPI-stained sections obtained from all the samples were analyzed, with a focus on the cartilage compartment. Several fluorescent nuclei (blue-dots) were recognized within NativeT lacunae; conversely, only few positive elements were detected into both DecellT and DecellT + CryoT specimens, which appeared comparable (Figure 2B). This evidence was further supported by DNA quantification. Across the entire cohort, average DNA content decreased from 131.87 ± 31.55 ng/mg in NativeT to 49.58 ± 13.18 ng/mg in DecellT and 44.54 ± 13.17 ng/mg in DecellT + CryoT, corresponding to reductions of ∼62% and ∼66%, respectively. Significant differences were observed when comparing NativeT to both DecellT (p < 0.01) and DecellT + CryoT samples (p < 0.001) (Figure 2C).

### 3.3 Histological appearance of trachea and structural proteins evaluation

**FIGURE 3:**
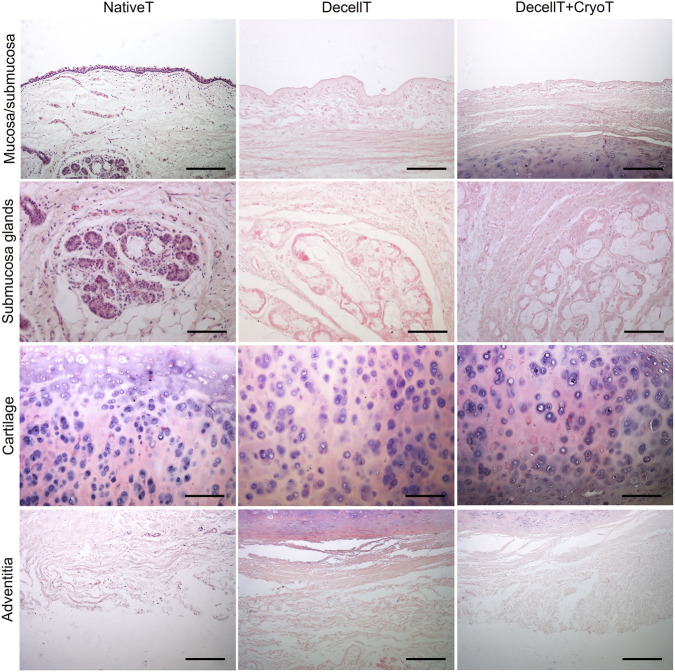
Histological characterization of native trachea (NativeT), decellularized trachea (DecellT) and decellularized trachea + cryopreserved trachea (DecellT + CryoT) by hematoxylin and eosin. Mucosa/submucosa, submucosa glands, cartilage and adventitia were focused individually and compared for overall morphology. Scale bar: 200 μm; submucosa glands, scale bar: 400 µm.

NativeT, DecellT and DecellT + CryoT samples were compared for their histological appearance focusing on the different compartments: mucosa/submucosa, cartilage and adventitia (Figure 3).

NativeT mucosa showed the typical complex organization of the respiratory epithelium with the ciliated pseudostratified columnar epithelium; conversely, only the thin basal lamina was recognizable following decellularization and decellularization + cryopreservation. The overall appearance of the submucosa was comparable in the whole cohort except for cell nuclei that were no longer detectable following decellularization and decellularization + cryopreservation.

**FIGURE 4:**
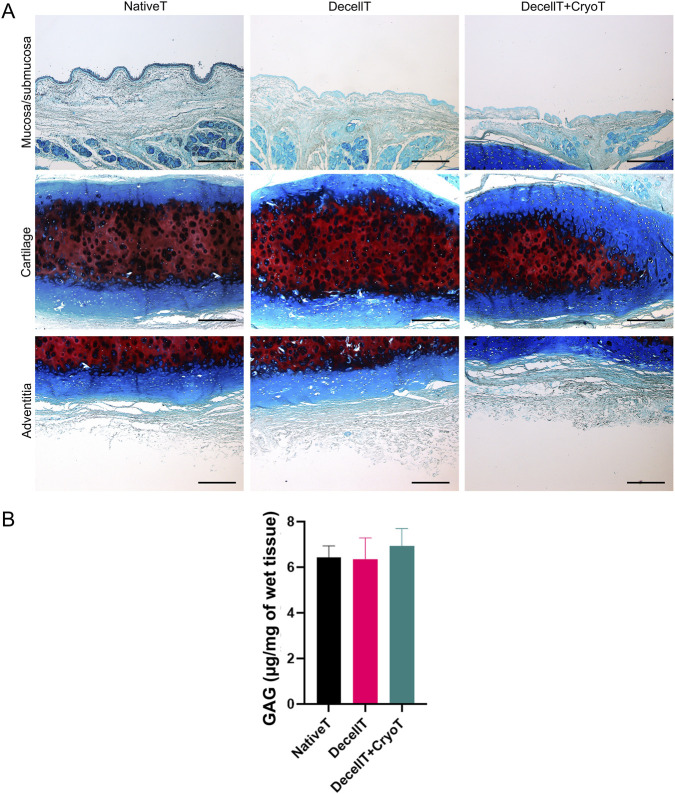
**(A)** Histological characterization of native trachea (NativeT), decellularized trachea (DecellT) and decellularized trachea + cryopreserved trachea (DecellT + CryoT) using Alcian Blue staining. Glycosaminoglycans (GAGs) are blue-stained. **(B)** Biochemical quantification of GAGs (µg/mg of wet tissue); no statistically significant difference was calculated within the cohort. Scale bar: 200 µm.

In parallel, GAGs distribution within the tracheal tissue was assessed. No significant variation in blue staining intensity was highlighted comparing the histological sections (Figure 4A). The same was later confirmed by biochemical quantification. Specifically, the NativeT samples showed an average GAGs amount of 6.435 ± 0.50 ug/mg which was comparable to that measured in DecellT (6.349 ± 0.94 μg/mg, ∼1.3% decrease) and DecellT + CryoT (6.941 ± 0.76 μg/mg, ∼7.9% increase), with no statistically significant differences between the groups (Figure 4B).

**FIGURE 5:**
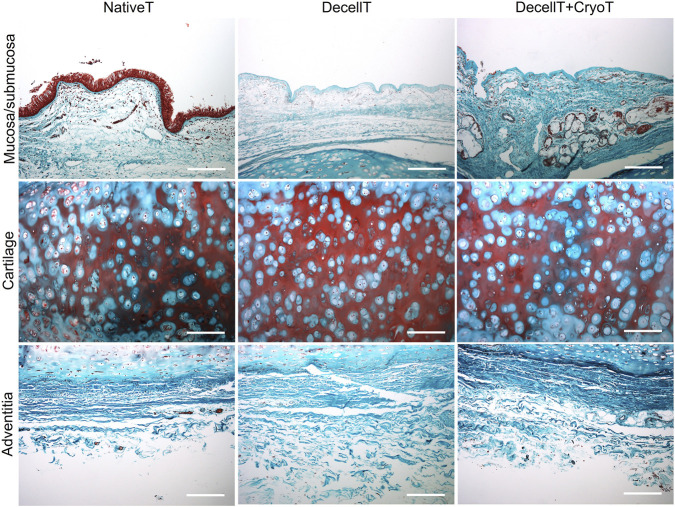
Histological characterization of native trachea (NativeT), decellularized trachea (DecellT) and decellularized trachea + cryopreserved trachea (DecellT + CryoT) by Masson’s Trichrome staining. Collagen is stained in green. Scale bar: 200 µm.

Collagen content was assessed by Masson’s Trichrome staining (green). No evident difference in colour intensity was observed, suggesting that the structural protein was maintained along with the treatments (decellularization and decellularization + cryopreservation). Distribution was fibril-like in both the submucosa and adventitia, while it appeared as more uniform within cartilage compartment (Figure 5).

**FIGURE 6:**
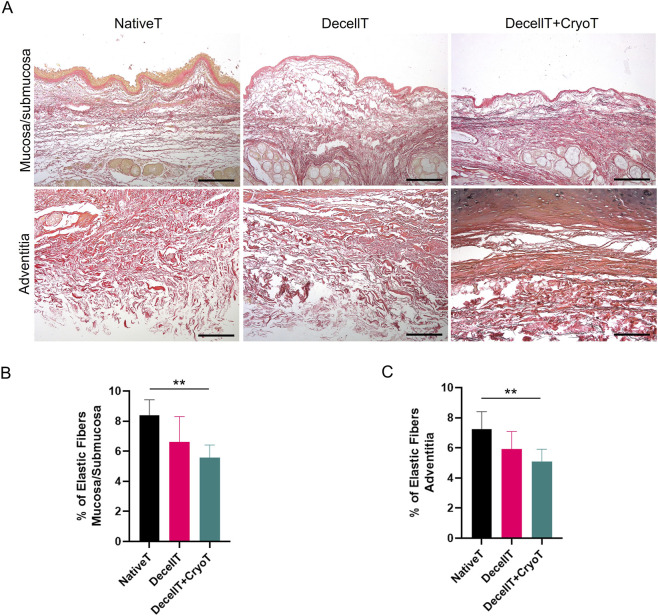
**(A)** Histological characterization of native trachea (NativeT), decellularized trachea (DecellT) and decellularized trachea + cryopreserved trachea (DecellT + CryoT) using Weigert Van Gieson staining; elastic fibers are stained in black. **(B,C)** Percentage of elastic fibers in mucosa/submucosa and adventitia (^**^p < 0.01). Scale bar: 200 µm.

Presence and arrangement of elastic fibers were evaluated in Weigert Van Gieson-stained sections. The fibers, which appear black, were recognized within the cohort and were localized in both the mucosa and submucosa and in the adventitia (Figure 6A). The highest elastic fiber content was observed in NativeT samples on both sides (mucosa/submucosa: 8.40% ± 1.02%; adventitia: 7.25% ± 1.15%), followed by DecellT (mucosa/submucosa: 6.62% ± 1.68%; adventitia: 5.94% ± 1.15%) and DecellT + CryoT (mucosa/submucosa: 5.57% ± 0.84%; adventitia: 5.10% ± 0.81%). Compared with NativeT, DecellT samples showed a reduction of ∼21% and ∼18% in the mucosa/submucosa and adventitia, respectively, whereas DecellT + CryoT exhibited a more pronounced decrease (∼34% and ∼30%, respectively). Moreover, cryopreservation further reduced elastic fiber content by about 16% in the mucosa/submucosa and 14% in the adventitia when compared with DecellT alone. Significant differences were detected between NativeT and DecellT + CryoT samples (p < 0.01) in both the mucosa/submucosa and adventitia (Figures 6B,C).

### 3.4 HLA-DR presence assessment and semi-quantification

**FIGURE 7:**
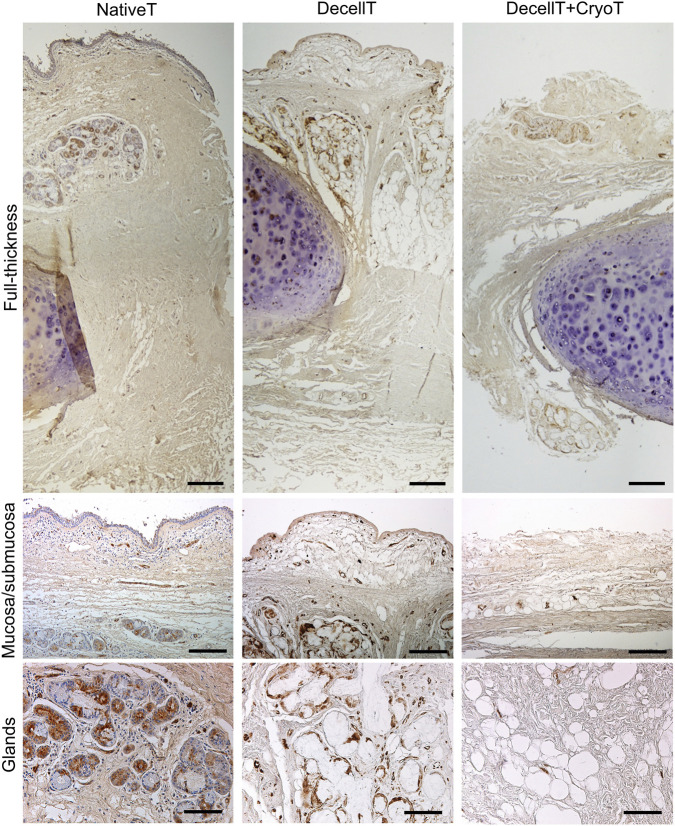
HLA-DR immunopositive elements detection in native trachea (NativeT), decellularized trachea (DecellT) and decellularized trachea + cryopreserved trachea (DecellT + CryoT); samples in full thickness, mucosa/submucosa side and glands were focused. Scale bar: 200 µm (full-thickness); 400 µm (mucosa/submucosa; glands).

Immunostaining enabled the detection and localization of HLA-DR–positive cells. In NativeT samples, immunoreactivity was primarily observed in the mucosa and submucosa, particularly associated with blood vessels and glands, along with a few isolated, brown-stained cells within the submucosal connective tissue. A noticeable reduction in HLA-DR–positive elements was observed in DecellT samples, with an even more pronounced decrease in DecellT + CryoT samples (Figure 7). Immunostaining assessments were confirmed by semi-quantitative analysis; the calculated DAB-positive area measured 6.7% in NativeT, decreased to 3.7% in DecellT, and was further reduced to 0.9% in DecellT + CryoT. A significant difference (p < 0.05) was detected between NativeT and NativeT + CryoT.

### 3.5 Ultrastructural characteristics of tracheal tissues

**FIGURE 8:**
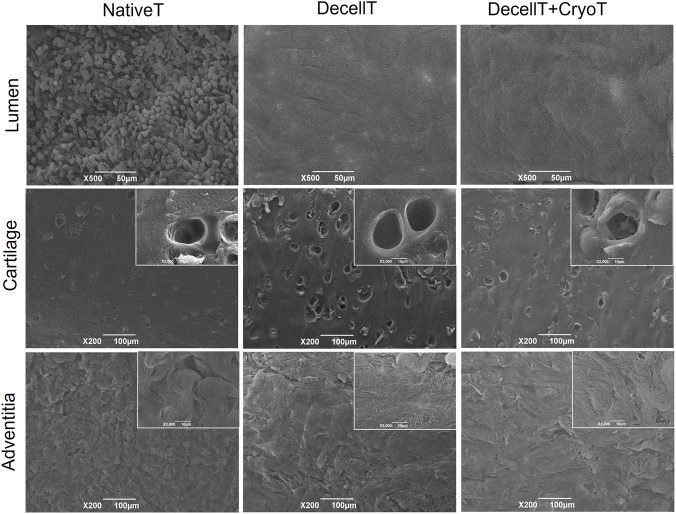
Trachea ultrastructure. Representative images showing ultrastructure of the respiratory epithelium, cartilage and adventitia layer in native trachea (NativeT), decellularized trachea (DecellT) and decellularized trachea + cryopreserved trachea (DecellT + CryoT) specimens. Scale bars: 500 µm and 100 μm; insert scale bar: 10 µm.

In accordance with SEM ultrastructural analyses, NativeT samples exhibited a epithelium rich in cellular elements, while the adventitia was characterized by wavy, orderly oriented, collagen fibers. The cartilage region showed the typical features of the hyaline cartilage, with the lacunae. DecellT and DecellT + CryoT samples were similar, both showed a marked reduction in the epithelial cells with maintenance of the basal lamina; in the adventitial layer, collagen fibers appeared as less compact/organized than in NativeT, forming a network. In the cartilage region, empty lacunae were detectable even at lower magnification (Figure 8).

### 3.6 Mechanical characterization

**FIGURE 9:**
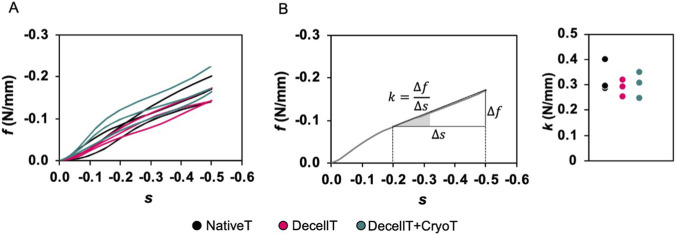
Results of compression tests. **(A)** Compression curve in terms of force per unit of length *f* vs. compressive deformation *s* in the proximal-distal direction. **(B)** The secant modulus *k* is calculated from each experimental curve as the slope of between the 20% and 50% of deformation (almost-linear region) and stiffness values are compared among the tested samples of native trachea (NativeT control), decellularized trachea (DecellT), decellularized + cryopreserved trachea (DecellT + CryoT). After treatments, not-significant variations of k are found compared with the control: in Donor 1, k increases in both DecellT (0.319 N/mm, +11%) and DecellT + CryoT (0.309 N/mm, +8%) relatively to the control (0.286 N/mm); in Donor 2, k decreases in DecellT (0.254 N/mm, −14%) and DecellT + CryoT (0.248 N/mm, −16%) with respect to the control (0.295 N/mm); in Donor 3, k decreases in DecellT (0.294 N/mm, −27%) and in DecellT + CryoT (0.350 N/mm, −13%) compared with the control (0.401 N/mm).

The compressive behaviour of NativeT, DecellT, and DecellT + CryoT samples is reported in terms of force per unit length as a function of tracheal compressive deformation in the proximal-distal direction (Figure 9A). A quasi-linear behaviour with increased stiffness was evident only after 20% of deformation, due to a possible variation of contact surface area during the test. A similar trend was previously observed also for porcine tracheal segments tested under compression by Stocco et al. (2023a) in the same experimental conditions. Overall, the compressive behaviour was comparable across all three experimental groups (Figure 9). The almost-linear region between 20% and 50% deformation was selected for the evaluation of the stiffness, with no statistically significant differences between the different groups (Figure 9B). The comparisons between NativeT and DecellT, NativeT and DecellT + CryoT, and between DecellT and DecellT + CryoT stiffness values yielded p-values of 0.95, 0.99, and 0.99, respectively, largely over the 0.05 threshold considered significant.

### 3.7 Cytotoxicity assessments

**FIGURE 10:**
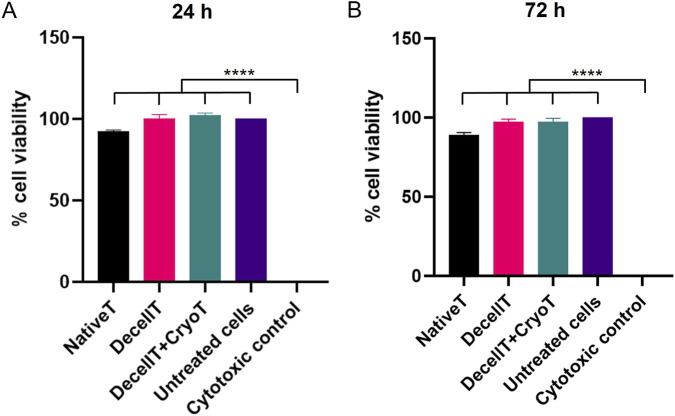
Cell viability of human bone marrow cells (HM1-SV40) grown in medium preliminary conditioned with decellularized trachea (DecellT) and decellularized + cryopreserved trachea (DecellT + CryoT). Non cytotoxicity of DecellT and DecellT + CryoT scaffold was confirmed by the preservation of >80% cell viability after culture. Untreated cells served as negative control; cells exposed to cytotoxic conditions were used as positive control. Two end-points were considered: **(A)** 24 h and **(B)** 72 h (****p < 0.0001).

At both experimental end points, HM1-SV40 cells appeared viable and actively proliferating. Approximately 40% and 90% confluence of the growth surface was reached after 24 h and 72 h of exposure, respectively. In accordance with the MTT assay results, cells maintained high viability when cultured in media conditioned with DecellT and DecellT + CryoT specimens. After 24 h of culture, cell viability was approximately 93% for NativeT, 100% for DecellT, and 102% DecellT + CryoT. After 72 h, viability values were approximately 89% for NativeT, 97.5% for DecellT, and 97.6% for DecellT + CryoT. At both time points, significant differences were observed between the cytotoxic control and all other experimental groups (p < 0.0001) (Figures 10A,B).

## 4 Discussion

The availability of clinical-grade, ready-to-implant tracheal grafts, manufactured according to the standards of an accredited tissue bank, may significantly enhance the surgical treatment of tracheal lesions (Arakelian et al., 2024). The encouraging clinical results obtained with cryopreserved aortic allografts (Makris et al., 2010; Martinod et al., 2022), demonstrate that tissue banking-based strategies can successfully translate into clinical practice even without decellularization. However, the non–airway-specific ECM requires a prolonged period of *in vivo* remodeling with stent support, and only partially resemble the native tracheal structure. Certainly, despite promising, intense efforts are still required to identify effective methods for accelerating *de novo* cartilage generation allowing early stent removal (Wei et al., 2024). In this perspective, recurring to decellularized tracheal scaffolds may represent a complementary and potentially more biomimetic strategy, particularly if combined with validated cryopreservation protocols compliant with regulatory standards.

In this work, decellularized human tracheal grafts (DecellT) were developed through a protocol specifically designed to ensure both safety and efficiency, while preserving the ECM. The grafts were then cryopreserved (DecellT + CryoT) and analyzed to assess the preservation of intrinsic characteristics. This evaluation provides preliminary information on the potential *in vivo* performance of the grafts, which is particularly relevant in the context of products intended for clinical application and required to meet the regulatory, safety, and quality standards of clinical-grade materials prepared by tissue banks. To date, as reported in the literature, intense efforts have been dedicated to the identification of an ideal strategy for trachea decellularization (Stocco et al., 2023a); often, despite obtaining a non-immunogenic substitute, it was observed ECM architecture disruption, with consequent issues *in vivo*.

Here, tracheal grafts were decellularized using a combination of hypertonic solution, benzonase, and sodium cholate. As observed with other tissues processed using the same protocol - such as pericardium and dermis (Montagner et al., 2024a; Montagner et al., 2024b) - a three-day treatment was enough to produce an acellular trachea. The residual DNA content measured was below 50 ng/mg of tissue, a threshold commonly regarded as indicative of a non-immunogenic scaffold (Crapo et al., 2011). Typically, reaching this level of DNA removal requires multiple cycles and longer treatments, or harsher methods like lyophilization (Stocco et al., 2023a). These strategies can affect not only tissue safety by leaving chemicals residues, but also negatively impact mechanical properties. For instance, the detergent Triton X-100, long used in many decellularization protocols (Kutten et al., 2015; Lange et al., 2017; Zhang et al., 2019), has been recognized as hazardous for the human endocrine system and was included within the European Chemical Agency (ECHA) list on 4 January 2021 (European Commission. Amending Annex XIV to Regulation (EC) No 1907/2006 of the European Parliament and of the Council Concerning the REACH, 2017) (European Commission, 2017). Sodium dodecyl sulphate (SDS), although effectively removing native tissue constituents and being broadly used in trachea decellularization protocols (for extensive review see Stocco et al., 2023a), is cytotoxic and requires intense washing to be removed (Alizadeh et al., 2019). Moreover, increasing SDS concentrations can alter structural proteins, resulting in elastic and viscous moduli reduction. Collagen fibers become compacted, and the content of GAGs, fibronectin, proteoglycans, and ECM regulators decreases, impairing not only cell colonization but also the retention of mechanical properties (Gilpin and Yang, 2017; Moffat et al., 2022). Stiffness reduction in the tracheal scaffold may be responsible of graft failure as consequence of deformation and airway obstruction (Gomes et al., 2025). In this study, sodium cholate was selected as the preferred detergent; to our knowledge, this is the first time that this trihydroxy bile salt is used for trachea decellularization. Even eventual combination with Triton X-100 in TRICOL was not reported. The choice of sodium cholate is further supported by its inclusion in decellularization protocols already approved by the *Italian National Transplant Center*, which may facilitate the potential translation of decellularized tracheal grafts toward actual clinical use. Alongside sodium cholate, enzymatic treatment with the exonucleases benzonase was performed. Benzonase degrades all forms of DNA and RNA in an efficient manner, without proteolytic activity, thus preserving ECM. Moreover, due to its low molecular weight, benzonase can readily penetrate tissue and can be also effectively removed through washing steps (Liu et al., 2019). Following treatment, the macroscopic appearance of the grafts was assessed. Both the DecellT and DecellT + CryoT grafts appeared whitish, with no noticeable differences in tissue color between the two groups. Moreover, unlike previous attempts in trachea decellularization, showing mechanical integrity compromission due to biochemical changes (Behrend et al., 2002; Partington et al., 2013; Stocco et al., 2023b), the DecellT samples remained firm, without collapse signs, thus suggesting preservation of airway patency. Similarly, the gross appearance of DecellT + CryoT grafts was comparable to that of DecellT, indicating that cryopreservation did not adversely affect their macroscopic integrity.

H&E staining confirmed the preservation of overall tissue architecture following decellularization. The decellularization protocol effectively removed the respiratory epithelium and cellular nuclei from the cartilage ECM, as well as from the mucosa/submucosa, cartilaginous compartment, and adventitia, without affecting the overall structural integrity and clear distinction of each tissue layer. DecellT + CryoT samples showed the same tissue organization of DecellT. The basal lamina was completely denuded but well-recognizable *in situ*: its presence is fundamental to guarantee re-epithelialization (Hegab et al., 2012). Airway epithelium complete regeneration is a complex phenomenon, in which interactions between epithelial cells and ECM play a crucial role. Once epithelial cells migrate in the area to be repaired, proliferation starts with the formation of a transitory squamous metaplasia followed by cells progressive redifferentiation that is a fundamental step to then restore the pseudostratified mucociliary epithelium (Coraux et al., 2008). Compact cartilage matrix was detected in the whole cohort suggesting that both decellularization and cryopreservation did not negatively impact over this structural layer, supporting macroscopic evidence.

The tracheal ECM is a highly specialized structure composed of collagen, elastin, GAGs, glycoproteins, and bioactive molecules that collectively determine the mechanical, structural, and biochemical properties of the organ (Gomes et al., 2025). While decellularization inevitably alters the ECM to some extent, effective protocols must achieve sufficient cell removal while preserving ECM ultrastructure and composition to maintain tissue architecture, minimize host immune response, and support cell behavior and tissue function post-implantation (Stocco et al., 2023a; Gomes et al., 2025). Similarly, cryopreservation, although essential for long-term tissue storage, can impact ECM integrity due to ice crystal formation and other stresses (Cunnane et al., 2021). Thus, this study employed histology, immunohistochemistry, biochemical assays, and ultrastructural analyses to evaluate the effects of both decellularization and cryopreservation on ECM preservation (Noro et al., 2024). Although ECM preservation is not a direct marker of immunogenicity, it is critical for ensuring graft biocompatibility and promoting constructive host remodeling (Romero et al., 2025).

GAGs are critical for maintaining graft structural integrity and bioactivity by supporting biomechanics and serving as binding sites for growth factors and chemokines involved in tissue remodeling and cellular colonization (Proudfoot et al., 2017; Smock and Meijers, 2018). Alcian Blue staining and biochemical quantification revealed no significant GAGs loss across native, decellularized, or cryopreserved grafts, indicating effective preservation of these key ECM components, despite tissue processing.

Collagen is the most abundant airway ECM protein with a primary role in determining airway mechanics (Liu et al., 2021). Different collagen types are distributed across tissue compartments: collagen type IV in the basement membrane; collagen type II is most abundant in airway cartilage, whereas collagen type I and III are responsible for mechanical airway properties. Through Masson’s Trichrome staining it was visualized the overall distribution of collagens. This staining also enabled differentiation between the fibrillar organization of the submucosa and adventitia and the denser, more compact structure of the cartilage layer characterized by the lacunae. Interestingly, no histological differences were detected between DecellT and DecellT + CryoT samples.

Elastic fibers, critical for tracheal flexibility and recoil (Harris, 1959), are predominantly located in the lamina propria and submucosa, arranged mainly longitudinally, with some oblique fibers forming a network (Kamel et al., 2009). Additional bundles are also observed in the adventitia (Andrade et al., 2011). Within the cohort, a reduction in elastic fibers presence and distribution was observed after both decellularization and decellularization + cryopreservation, likely due to greater exposure to the chemical solutions used during processing; however, no impact over mechanical behaviour was identified.

Although tracheal cartilage is often considered an “immune-privileged” tissue (Stocco et al., 2023a), the mucosa is highly antigenic and represents the main source of graft immunogenicity. In particular, the airway epithelium and submucosal glands exhibit strong immunoreactivity to the HLA-DR antigen (Bujia et al., 1990). Therefore, effective decellularization must eliminate these immunogenic components. Previous morphological evaluations confirmed the absence of ciliated epithelial cells and glands in both DecellT and DecellT + CryoT samples. Consistently, no HLA-DR–positive elements were detected post-treatment, suggesting a possible reduction in graft immunogenicity and an immunological profile that would be favorable for implantation. Ultrastructural characterization can help determine whether the decellularization protocol was effective in achieving a satisfactory degree of tissue decellularization; additionally, it can reveal any modifications to the ECM network (Maughan et al., 2017; Sun et al., 2021; Zhang et al., 2022; Liu et al., 2021). In NativeT samples, the respiratory epithelium was clearly distinguishable. Post-decellularization, this layer was absent, exposing an intact basal membrane. The same pattern was observed in DecellT + CryoT samples, indicating that cryopreservation did not induce further structural alterations. Additionally, empty lacunae were visible following decellularization and maintained their appearance after cryopreservation. Regarding the adventitia, collagen fibers in the NativeT were arranged in bundles, but this organization was partially altered by the decellularization treatment, leading to a more network-like structure.

Assuring for suitable mechanical properties in DecellT and DecellT + CryoT samples is critical for their clinical application. The graft must preserve native compressive stiffness and structural integrity to withstand respiratory forces and ensure airway patency. Excessive softening could lead to deformation, collapse, or poor integration with host tissue. In the present study, despite a limited sample size, no statistically significant differences in the compressive stiffness were found among NativeT, DecellT, DecellT + CryoT, thus indicating suitable mechanical properties for application of DecellT and DecellT + CryoT graft *in vivo*. The biochemical modifications did not compromise mechanical integrity. Previous studies on the mechanical properties of human (Safshekan et al., 2017) and animal (Huang et al., 2021) tracheal tissues have reported that stiffness is not significantly influenced by the sample location along the tracheal duct; however, regional variations above 50% between the stiffest and most compliant tracheal regions have been documented. Accordingly, the modest stiffness variations observed in the present study (below 30%) are more likely attributable to intrinsic tissue heterogeneity rather than to the applied treatment.

Cryopreservation is the best option to preserve tissues over time, avoiding the onset of degradation phenomenon likely associated with consequent graft failure. However, it can damage the tissue due to ice crystal formation; it descends that the identification of an ECM-respectful protocol is fundamental (Cunnane et al., 2021). Structural damage not only impairs mechanical properties but can also hinder cellular integration and tissue regeneration post-implantation (Gilpin and Yang, 2017).

## 5 Conclusion

To date, decellularized tracheal matrices are considered an ideal scaffold for regeneration (Xu et al., 2017); however, along with difficulties in the identification of an effective protocol for donor’s cells removal, long-term preservation of these grafts, assuring for morpho-structural characteristics maintenance, is debated.

The decellularization protocol developed by the FBTV, recurring to a combination of hypertonic solution, sodium cholate, and benzonase, was shown to reduce immunogenic components from human tracheal grafts while preserving fundamental ECM structural components, including collagen, glycosaminoglycans, and elastic fibers. This preservation ensured the maintenance of graft integrity and mechanical properties. Retaining histological features is fundamental for re-epithelialization and graft function. In parallel, also cryopreservation impact over DecellT was assessed. Cryopreservation did not induce DecellT damage or degradation, as confirmed by macroscopic, histological, ultrastructural and mechanical analyses. Further, a decrease in DNA content and HLA-DR antigens was observed in DecellT + CryoT compared to both NativeT and DecellT. No heterotopic or orthotopic implantation in animal models was performed, which represents a limitation of the study; moreover, a more comprehensive immunological characterization would be warranted, including the assessment of HLA class I molecules (HLA-A, HLA-B, HLA-C) and co-stimulatory molecules (CD80, CD86, CD40), as well as functional assays (e.g., mixed lymphocyte reaction and IFN-γ ELISPOT), to better define the immunogenic profile of the grafts. Taken together, the findings suggest that the DecellT protocol may reduce immunogenic elements, and that cryopreservation may further enhance these properties.

The protocol used in this study was previously validated on other tissue types already approved by the *Italian National Transplant Centre* for clinical application, demonstrating compliance with regulatory requirements for the processing of human tissues intended for clinical use. These requirements, including quality management, traceability, environmental controls, and mandatory sterility testing, were also successfully fulfilled here, in accordance with applicable regulatory standards.

The number of tracheal samples included in this study is limited, representing a potential constraint; however, this reflects intrinsic regulatory limitations. Human tracheal tissues were obtained exclusively from the Fondazione Banca dei Tessuti del Veneto and the *Italian National Transplant Centre* authorizes their use for validation purposes only when they are deemed unsuitable for transplantation. Considering that tracheal tissue is rare and only a small number of tracheas become available for non-clinical use, the sample size is unavoidably restricted.

Overall, in the light of the study results, cryopreservation of DecellT enables long-term storage without compromising tissue integrity, thus supporting this strategy for further investigation. This approach may offer safe, mechanically stable, and ready-to-implant tracheal scaffolds, representing a significant advancement in airway tissue engineering. Although further evaluation through robust preclinical studies is required to assess the biological, morpho-structural, functional, and mechanical properties of the scaffolds following heterotopic and orthotopic implantation in animal models, these initial findings are encouraging with respect to future clinical translation.

## Data Availability

The original contributions presented in the study are included in the article/supplementary material, further inquiries can be directed to the corresponding authors.
